# ASAS-NANP SYMPOSIUM: prospects for interactive and dynamic graphics in the era of data-rich animal science^1^

**DOI:** 10.1093/jas/skaa402

**Published:** 2021-02-24

**Authors:** Gota Morota, Hao Cheng, Dianne Cook, Emi Tanaka

**Affiliations:** 1 Department of Animal and Poultry Sciences, Virginia Polytechnic Institute and State University, Blacksburg, VA; 2 Center for Advanced Innovation in Agriculture, Virginia Polytechnic Institute and State University, Blacksburg, VA; 3 Department of Animal Science, University of California, Davis, CA; 4 Department of Econometrics and Business Statistics, Monash University, Clayton, VIC, Australia

**Keywords:** dynamic graphic, human–computer interaction, image, interactive graphic, statistical graphic, visualization

## Abstract

Statistical graphics, and data visualization, play an essential but under-utilized, role for data analysis in animal science, and also to visually illustrate the concepts, ideas, or outputs of research and in curricula. The recent rise in web technologies and ubiquitous availability of web browsers enables easier sharing of interactive and dynamic graphics. Interactivity and dynamic feedback enhance human–computer interaction and data exploration. Web applications such as decision support systems coupled with multimedia tools synergize with interactive and dynamic graphics. However, the importance of graphics for effectively communicating data, understanding data uncertainty, and the state of the field of interactive and dynamic graphics is underappreciated in animal science. To address this gap, we describe the current state of graphical methodology and technology that might be more broadly adopted. This includes an explanation of a conceptual framework for effective graphics construction. The ideas and technology are illustrated using publicly available animal datasets. We foresee that many new types of big and complex data being generated in precision livestock farming create exciting opportunities for applying interactive and dynamic graphics to improve data analysis and make data-supported decisions.

## Introduction

Graphics in science are visual representations often employed to illustrate the concepts, ideas, or outputs of research. Some examples of graphics include photography, schematics, diagrams, flowcharts, and images. In particular, statistical graphics, a subfield of data visualization, are used to display patterns in data, communicate the results of data analysis, and help diagnose numerical techniques. Scatter plots, histograms, bar charts, and box plots are representative examples of statistical graphics. Statistical graphics focuses attention on the display and understanding of uncertainty, which is not necessarily the emphasis broadly in data visualization.

Statistics has been fundamental to animal science research and animal science curricula (Street, 1990; Nelsen, 2002). However, little attention has been paid to the importance of graphics. As data collections become more detailed and complex, generating larger data sets and more complicated experimental designs, the use of graphics along with numerical techniques becomes increasingly important. Technology has matured to make it easier to construct and share graphics. The availability of modern web browsers coupled with improved computer hardware promoted developments of tools to construct interactive and dynamic graphics.

The interpretation of the terms “interactive” and “dynamic” can vary and has potentially changed as technology evolved (Swayne and Klinke, 1999). Here, a graphic will be called “interactive” if users can interact with the graphic through user interaction such as hovers or clicks. Note that Swayne and Klinke (1999) refer to this as “direct manipulation”. A graphic is “dynamic” if the graphic updates its content in real-time, for example, a 3D rotation. Following Swayne and Klinke (1999), “indirect manipulation” means the manipulation of graph by graphical user interface controls such as a slider.

Regardless of the type of data graphic, there are many purposes: exploratory, model support, and for communicating results. Graphics for exploratory data analysis need to be quick to produce, and can be rough, to allow for the data to inform what was not known. Model support graphics are used for checking assumptions and for checking the fit. Communication graphics need to be designed to optimally present the important information, and to have an attractive appearance.

In animal science, there has been many new types of data collection techniques adopted recently. For example, with the advancement of precision livestock farming has come the availability of large, detailed, and complex data types, including sensor-based image data delivered in real-time. This new, large, and complex data provides fresh challenges in developing and managing graphics for data analysis in animal science (Morota et al., 2018; Koltes et al., 2019).

The objective of this review article is to describe the state of the field of interactive and dynamic graphics that can be applied in animal science problems. We also provide some conceptual framework underlying the tools, as seen through a statistical graphics lens of exploring variability and uncertainty. Publicly available animal data are used for illustration purposes. Beyond statistical graphics, we also discuss interactivity in multimedia (text, images, videos, and computer graphics) in brief. Although there is visualization capability available in many programming languages, this article focuses on those readily available in R (R Core Team, 2020) and partially in Python (Van Rossum and Drake Jr, 1995), 2 leading (open-source) languages in data science. When referring to a programming library or package, an object or a function, we write in monospace font throughout the article.

This article is organized as follows: The next section describes data examples used later and general data structures to expect or create for effective visualization. The data visualization section provides overview frameworks for thinking about statistical graphics. This is followed by a section describing methods for interactive graphics and the current technology available. The interplay between new data types, data visualization, and machine learning is also described. The conclusion discusses where we expect the field to grow in the coming years.

## Illustrative Data

### Data sources

In this section, we briefly describe the data used to illustrate concepts in statistical graphics throughout the paper. More details of each study can be found in the respective references.

#### Quail

The experiment by Sarraude et al. (2020) investigated the effects of prenatal thyroid hormones injections to quails on a number of traits (e.g., egg hatching success, embryonic development, and offspring growth). The eggs were injected with the prohormone ($T_{4}$), its active metabolite ($T_{3}$), a combination of both $T_{3}$ and $T_{4}$ ($T_{3}T_{4}$), or none (i.e., control eggs, CO). The trial involved 21 mother quails with a total of 57 offspring belonging to one of the four treatments groups ($T_{4}$, $T_{3}$, $T_{3}T_{4}$, or CO).

#### Sheep

Posbergh and Huson (2021) conducted a genomic investigation to mature body size of sheep. The data contain various body measurements of 217 ewe of various sheep breeds. These ewes are genotyped with 506,939 single-nucleotide polymorphism markers.

#### Chick

The body weights of 50 chicks were measured across 12 time points after their birth to investigate the effect of 4 different diets. These data were an example by Crowder and Hand (1990) and can be found in the datasets package in R (R Core Team, 2020).

#### Cow

Genome-enabled complex trait prediction was carried out by Gianola et al. (2011) using Bayesian neural networks. The dataset contains milk, protein, and fat yields of 297 Jersey cows, their corresponding genomic relationship matrix constructed from 35,798 markers, and the number of lactations. The dataset is available in the study by Pérez-Rodríguez et al. (2013).

#### Lamb

The lamb dataset included birth weight and weaning weight of 882 lambs from a partial diallel cross of Dorper and Red Maasi breeds as a part of parasites resilience experiment at the International Livestock Research Institute in Kenya from 1991 to 1996 (Baker et al., 2003). The dataset is available in the agridat R package (Wright, 2020).

### Data structures

Although data visualization should be one of the first key steps in data analysis, certain data structures can hinder this process, thus the data may initially need to be wrangled to a suitable format. While the exact data structure depends on the downstream visualization tool, the concept of tidy data (where each variable is a column, each observation is a row, and each value has its own cell; see more details in the study by Wickham, 2014) greatly facilitates the use of grammar of graphics (described later) to make effective plots.

Recent data collection methods have spawned a new type of data structure where the original data are images (Fernandes et al., 2020b). Examples include the computed tomography scan of carcasses (Navajas et al., 2010), thermal imaging (Scoley et al., 2019), and depth images (Kongsro, 2014) and more examples under the Computer vision section.

## Data visualization

Empirical studies require investigators to extract meaningful information from the data. While appropriate statistical inference has been instrumental for confirmatory analysis, numerical summaries or significance tests can be reductive in forming a fuller understanding of the data and in many cases requires rigorous understanding of statistics for appropriate interpretation. Data visualization has a broad role in data analysis from initial examination of data (Chatfield, 1985) to visual inference (Buja et al., 2009) and complements any numerical summaries. Well-chosen statistical graphics make well use of the human visual system and give insight to data, even to the less statistically trained individuals.

Data visualization has long been used for viewers to see patterns, trends, or anomalies (Friendly, 2008) and continues to be instrumental in the era of big data (Cook et al., 2016). Not all data visualization is made equal in its ability to communicate information to the viewer. Cleveland and McGill (1984) conducted empirical experiments to determine the order of accuracy for viewers to extract the numerical value from statistical graphics with certain graphical elements, e.g., they found that viewers are more accurate in evaluating the values of lengths (say in a bar chart) compared with reading colors (say in a heatmap). Zeileis et al. (2009) discusses the use of color in statistical graphics and advocate the use of HCL-based color palettes. Carefully chosen graphical elements enhance capabilities of statistical graphics to communicate information from the data, and we illustrate some of these elements with examples in later sections.

This section is organized as follows: we first discuss the core principles of the grammar of graphics implemented in the ggplot2 R package (Wickham, 2016), which is used to create all static graphics in this article with code to reproduce all graphics is in the link provided under the Code section. We then discuss the use of statistical graphics in 4 aspects of statistical analysis illustrated with the study of quails and the study of sheep genetics (see the Data sources section for descriptions). We acknowledge that there are many other uses of data visualization in the animal science field; however, we cannot cover the entirety of the field in this article alone, so the examples are not an exhaustive representation of all data visualization in animal science.

### Grammar of graphics

Just as the genetic footprint of an organism allows us to comprehend the genetic characteristics of the organism, in order to better understand statistical graphics, we require the notion of the *grammar of graphics* (Wilkinson, 2005) to dissect statistical graphics to its core components. As an analogy, consider livestock such as cow, sheep, and goat. Cow, sheep, and goat are all distinct species with characteristic genetic composition for its own species, yet each species share some common genetic functions observable only at the genetic level. Now consider statistical graphics such as pie chart, bar plot, and rose plot—each indicative of a particular type of plot, yet each plot may depict the same information from the same set of data. The named statistical graphics, much like animal names, is a contraction that makes everyday communication easier but can obscure cognitive process to see the relation between plots or to create new type of plots.

The grammar of graphics describes the syntax and semantic specifications to compose a statistical graphic which interplay as the building blocks of an object-oriented graphical system. This flexible system was later extended and implemented as the ggplot2 R package (Wickham, 2016). The core system constructs graphical layers with each layer consisting of a tidy data, a geometric object (e.g., point and line), a statistical transformation (e.g., identity for a scatter plot or a five number summary for a boxplot), mapping of data variables to aesthetic attributes (e.g., color, size, and x- and y-coordinates), and a positional adjustment for overlapping objects. Furthermore, the system allows facetting by a categorical variable, where facetting refers to multiple panels of the same plot with each plot using a subset of the original data as indexed by the categorical variable. Complex statistical graphics can be composed from these layers build on this coherent system. This system can be extended by other authors and there exists numerous extensions, some for constructing specialized plots, including the ggbio Bioconductor R package (Yin et al., 2012) used for visualizing genomic data.

The impact of ggplot2 is evident from it accruing over 24,000 citations, the adaptation of its system to Python as the plotnine library (Kibirige et al., 2020) and is the graphics system of choice for BBC Visual and Data Journalism (2019). Why is it so well utilized? There are identifiable benefits of using a programmatic approach that can build a variety of complex plots. These benefits include reproducibility and transparency but because it is built on a coherent system, the user does not have to recall specialized or ad hoc approaches for every complex plot they wish to construct.

### Comparative studies

The basis of many quantitative studies lies in comparison; this could stem from comparison between treatments, states, distribution, or other variables of interest. These comparisons are generally carried out based on statistical models (this includes the basic comparisons of means of 2 treatment groups using the t-test (Student, 1908), which can be formulated from testing the treatment contrast of a linear model with the treatment effect); however, the formulation of these models often require visualizing and exploring the data.

For the remainder of this section, we examine the quail data to illustrate treatment comparisons with graphics. In the quail data, one of the interests is to compare the effects of treatments on body mass; however, the body mass is a function of age. Do we compare treatments at a specific age? Or should we base this comparison on the differences of body mass at two time points? Do we assume that the treatment and age effects are additive? Comparing treatments are not straightforward when there are peripheral variables that affect the response of interest. Data visualization plays an important role to formulate statistical approaches to make these comparisons.

**Figure 1.**
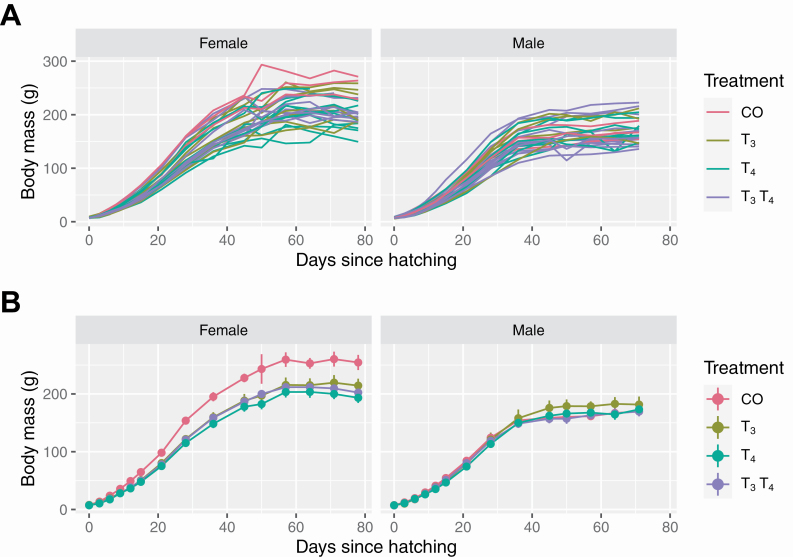
Plot A: the line graph of body mass in grams over age in days for each offspring with line colored by the treatment group and plot facetted by sex of the offspring. Plot B: The points and line graph in the above figure show the average body mass over age by the sex of offspring. The vertical lines through the points show the standard error of the mean.

How should we visualize the body mass data? As the body mass is a function of age, we can consider creating a line graph for the body mass over age with each line corresponding to a single quail offspring; this produces a plot what is commonly referred to as a spaghetti plot. However, the data contain 57 quail offspring, so it is difficult to disentangle the “spaghetti” in the plot. As sex is generally a large contributing factor to determine body mass, splitting the data by sex helps reduce the spaghetti in one plot (here we have 29 females and 28 males), but this is still hard to follow as seen in Figure 1A. A common approach instead is to plot the statistical summaries, such as the mean of each treatment group, with uncertainty measures depicted with an error bar (Figure 1B) but any individual characteristics are lost (e.g., one female quail in the CO group has a sizable increase in their body mass at 50 d old as seen in Figure 1A, and this is contributing to the large uncertainty in Figure 1B).

**Figure 2.**
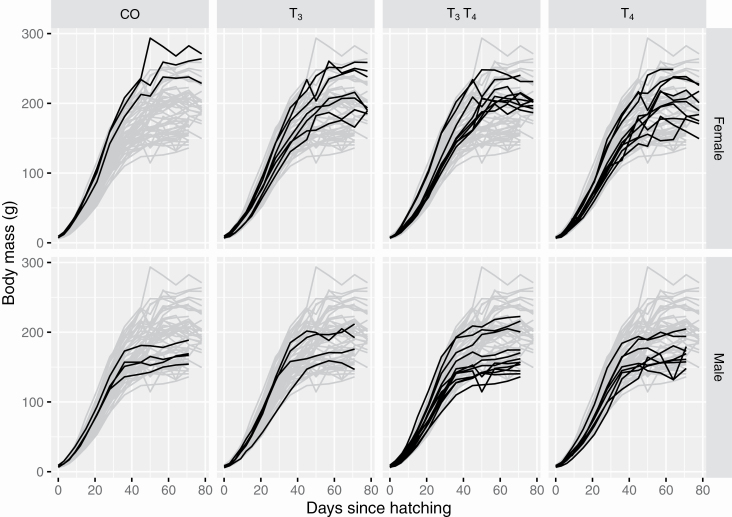
The black points and lines show the body mass in grams over age of the offspring of the quails. Each curve corresponds to one quail offspring. The gray curves correspond to the observations of all quail offspring disregarding the treatment group or the sex. The growth curve is facetted by the treatment group and sex of the offspring.

Alternative compromising approach to visualize these data is to select a subset of units to visualize at a time and there exists tools, such as the brolgar R package (Tierney et al., 2020) and interactive data visualization tools discussed further in the Interactive graphics section, to make the construction easier for the user. Since there are only 3 to 12 replicates for each treatment group and sex combination, we can visualize these data facetting by these groups as shown in Figure 2. There are a number of graphical techniques used to enhance Figure 2 including: (i) the treatment comparisons are facilitated by the use of common scales, and (ii) the grey lines shadow the growth of all individuals regardless of treatment and sex group. The use of common scales was the top-ranked elements in the empirical experiment by Cleveland and McGill (1984) for viewers to extract accurate numerical information from statistical graphics. The use of shadows assists highlighted lines to be directly compared with the rest of the data, e.g., we can easily see the slowest growing individual is a male in the $T_{3}T_{4}$ treatment group.

### Visualizing statistical models

Statistical models are commonly used in the analysis with the goal of conducting inference in animal science, e.g., understanding the effectiveness of a treatment to the trait of interest of an animal. The use of complex models, such as mixed models (also known as multi-level modelling or hierarchical modelling) and generalized additive models, is rising in prominence with wide availability of software, such as the lme4 (Bates et al., 2015) and mgcv (Wood, 2017) R packages, and the increase in collective community knowledge about these models. Though there is more software capability to fit complex models, the interpretation and understanding of the model is not readily done, or in some cases misinterpreted even by statistical experts. Diagnostics and interpretation of statistical models can largely be assisted by the use of data visualization.

Model selection is an active research area and we only briefly describe here the role of data visualization in constructing appropriate statistical models. Before proposing any candidate models, it is most sensible to visualize the data. The purpose of data visualization here is multifaceted: the user may realize that data may require transformation; the user may notice that there are some unusual observations that may need to be further queried; or the user may see that the relationship between pairs of variables are nonlinear. From Figure 2, we can see a clear nonlinear relationship between age and body mass of a quail, and the growth curve is noticeably different between the treatments groups and sexes, with females generally being bigger than males after 40 d of age. Given these observations, we would think that the proposed model for mass should contain sex and treatment with a nonlinear relationship with age.

Sarraude et al. (2020) fitted a number of candidate models to the growth in body mass of quails using a nonlinear generalized additive mixed model. For illustration, we consider the model, henceforth referred to as the generalized additive model, that incorporated a temporal autocorrelation of the residuals using an autoregressive moving average ARMA(1, 1) model; a random intercept for the mother’s identity; treatment group, and sex as fixed effects; and thin plate regression spline for the age of the offspring varied by sex. The model is fitted using the mgcv R package and the code is available in the link under the Code section.

**Figure 3.**
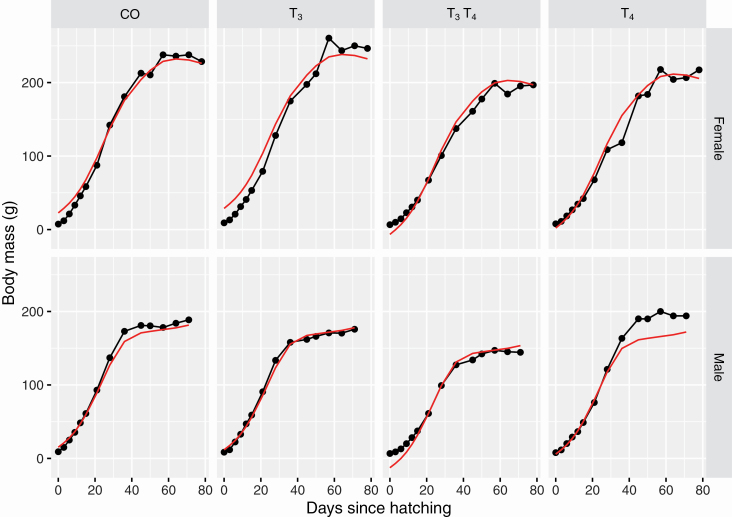
The black lines and points show the body mass over time of a sample of 8 offspring, one each from the combinations of treatment group and sex. The superimposed red line is the predicted growth curve from the generalized additive model. The number in the background of each facet is the egg ID of the quail.

Any inference or prediction based on this model should only be followed after performing model diagnostics. Generally, these diagnostics involve analysis of the residuals (the difference between the observed and predicted value); however, residual analysis alone offer a restrictive perspective. An alternative approach is to display the model in the data space (Wickham et al., 2015) where it is easier to see where the model may or may not be performing well. Figure 3 shows the predicted values of quail mass based on the generalized additive model for a sample of 8 quails, and we can see that the model fits fairly well for those shown except for 1 with egg ID 224 where the predicted body mass is lower than the actual body mass for later in its life. Figure 3, however, only samples 8 quails and further exploration can be facilitated by interactive graphics where the user may interactively select the quails they wish to observe.

### Visual inference

Statistical inference, such as the frequentist framework where a hypothesis is tested using a (numerical) test statistic under its null distribution, generally follows a rigorous procedure to draw conclusions based on values such as *P*-value, critical values, or confidence intervals. This testing framework is widely utilized; however, there is generally a lack of rigorous procedure for drawing inference from statistical graphics.

**Figure 4.**
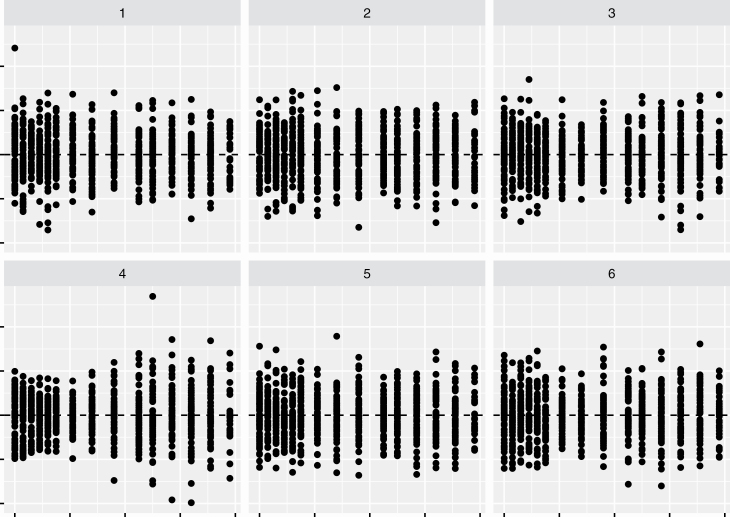
One of these plots is the residual plot from the fit of the generalized additive model to the actual data and the others are the residual plot from the fit of the model to the null data. Which plot looks the most different?

Consider the use of residual plots (i.e., a scatter plot with the residuals against the predictor or fitted value) to search for any remnants of missing terms in the model. An identifiable pattern in residual plot is an indicator of missing terms in the model that explain the response. The residual plot is often judged in isolation but a more formal, rigorous approach will calibrate this plot using a visual inference framework (Buja et al., 2009). In this visual inference framework, a lineup such as Figure 4 is shown to a number of viewers where one plot is the actual residual plot and others are residual plots constructed from data under a pattern that is consistent with randomness or a known model. These plots are referred to as null plots. The context of the plots is removed as not to bias the viewers and the viewers are asked to identify a plot that looks most different to them. If the viewer’s choice appears random, this means that the actual residual plot does not have identifiable feature that stand out from the null plots.

Visual inference is a formal procedure to make inference from statistical graphics, and unlike classical inference the (numerical) test statistic is a data plot. The actual data plot in Figure 4 are the fourth one. If many of you identified this plot, this means that there is some identifiable pattern in this residual plot that distinguishes it from null plots, thus the generalized additive model may not be adequately fitting the data. Conversely, if the fourth plot was chosen just as many other times other plots, then there is no evidence to indicate that the data plot is any different to null plots and thus the model may be adequate to explain the data.

### Visual narrative

A statistical graphic is constructed with an aim to communicate about the data to the viewer. Depending on the audience, the graph may be polished to suit its end purpose, e.g., labeling legends and scales appropriately. A polished up graph, however, can be further enhanced to direct viewers to the narrative of the data, e.g., annotating or highlighting the trait of interest. This enhancement should not come at a cost to hide or distort the information from the data. We show an example using the Manhattan plot next.

**Figure 5.**
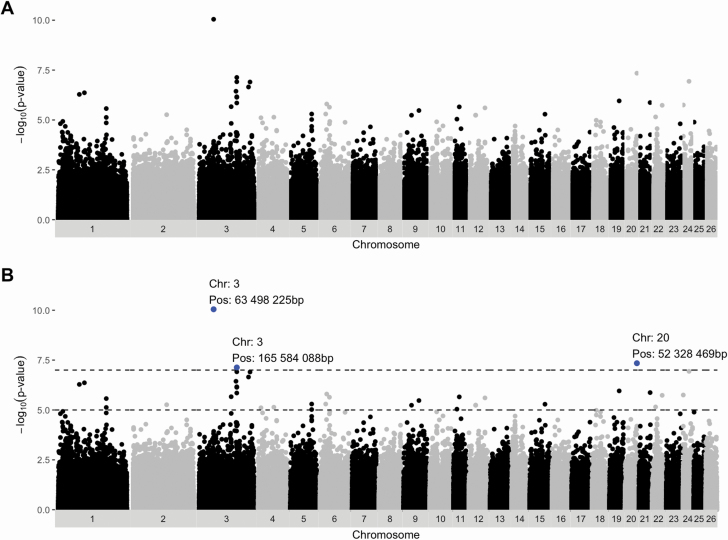
Both Manhattan plots show $-log_{10}(P\text{-}value)$ of the genome-wide association analysis of withers height of sheep. Plot A is an informative plot while Plot B has extra annotations that enrich the narrative about the genome associations with the trait of interest. The horizontal dashed lines indicate where $-log_{10}(P\text{-}value)$ is 5 and 7.

In a genome-wide association study, it is common to display a Manhattan plot of $-log_{10}(P\text{-}value)$ as shown in Figure 5. The *P*-values in Figure 5 are calculated from the genome-wide association analysis based on testing each single-nucleotide polymorphism markers. More specifically, a marker is fitted as a fixed effect in a mixed model with animals is fitted as random effects with multivariate Gaussian distribution with mean of zeroes and covariance proportional to the genomic relationship matrix (Hayes et al., 2009). The model fitted is equivalent in the study by Yu et al. (2006) or EMMAX described by Kang et al. (2010) and calculated based on the rrBLUP R package (Endelman, 2011).

The typical goal of these Manhattan plots is to display the significant and nonsignificant genetic markers with respect to its chromosomal position. While Figure 5A is sufficient as an *informative plot*, extra annotations in Figure 5B highlight the significant markers according to a cut-off of $-log_{10}(P\text{-}value)$ at 7. This latter plot is more powerful in conveying the narrative about potential genetic associations to trait of interest.

## Interactive Graphics

**Figure 6.**
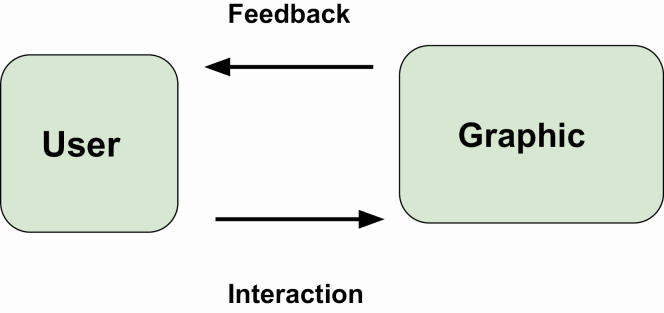
Scheme of interactive graphics. The user interacts with the graphic by hovering or clicking. The graphic returns feedback to the user.

Interactivity facilitates human communication. A speaker talks to a listener. The listener responds. The speaker then returns to the listener. This constant back and forth is essential for conversational dialog. The same concept applies to data communications. First, a human controls or sends a signal to the point of interest in a visual representation. The visual representation promptly responds to the human input and changes its visual output in a timely manner. One example is a user directly hovers or clicks an interactive graphic being presented. The interactive graphic then gets back to the user showing the specific information in the graphic the user requested. When the user hovers or clicks a different location of the graphic, the interactive graphic returns an updated rendering to the user (see Figure 6 for an example). Graphical user interfaces are designed to provide meaningful responses to mouse hovers or clicks. Akin to human communication, this constant communication feedback loop between the user inputs and the graphical responses facilitates data communications and is what sets interactive graphics apart from static ones. Data exploration is where interactive data visualization shines. Interactive graphics efficiently enhance human–computer interactions by mining signals and patterns from the data. Unwin (1999) stated that direct querying, zooming, rescaling, selection with linking, and the use of multiple views should be presented by interactive graphics software for exploratory data analysis.

Undeniably, the best way to deploy interactive graphics is on a web page using cascading style sheet or JavaScript. The advancement of web technologies (e.g., HTML5) and the availability of modern web browsers have broadened the dissemination of interactive graphics. User-friendly web interfaces to JavaScript libraries are available in both R and Python, providing good options for data visualization. Interactive graphics are often web-based and interweaved into web documents. Interactive data visualization, for example, is trending with major online newspapers (e.g., New York Times, Washington Post, and Financial Times; Hohman et al., 2020). Interactive graphics are likely to become ubiquitous among other online publications, including scientific articles. This is important for journals, and the movement is burgeoning in the area of machine learning. For example, *Distill* is a peer-reviewed journal that promotes explorable explanations using interactive figures and tables for machine learning research (https://distill.pub/). Some papers published by *Distill* include interactive visual exploration of Gaussian processes (Görtler et al., 2019) and Bayesian optimization (Agnihotri and Batra 2020), which are actively investigated to enhance (Momen et al., 2018) or optimize (Tanaka and Iwata 2018) genomic selection.

### Interactive statistical graphics

Interactive graphics are especially useful for statistical representations. The limitations of static graphics vs. the advantages of interactive graphics can be found in the literature dating back to the 1960s (Becker et al., 1987). John W. Tukey, who is known for Tukey’s test or Tukey’s honest significance test (used for animal science research), has made many contributions to interactive statistical graphics by promoting exploratory data analysis (Tukey and Tukey 1988; Friedman and Stuetzle 2002). Tukey was, for example, involved in the development of PRIM-9: the first interactive and dynamic graphic system to analyze multivariate data (Fisherkeller et al., 1974). A video demonstrating the use of PRIM-9 is available at the American Statistical Association Section on Statistical Computing and Statistical Graphics (http://stat-graphics.org/movies/prim9.html).

GGobi (Swayne et al., 2003) is an interactive data visualization software for exploratory data analysis. It is a descendant from DataViewer (Buja et al., 1987), XGobi (Swayne et al., 1998), and Orca (Sutherland et al., 2000), which were developed after PRIM-9. GGobi implements the concept of multiple linked plots (Buja et al., 1991), which allow users to generate multiple linked views of the same data in different interactive graphs (e.g., scatter and bar plots) (Cook and Swayne 2007). For example, if the user mouses over a certain data point in a scatter plot, the corresponding area in the bar plot will be highlighted simultaneously as the same data object is modified. Linked views are often used in *brushing* (Becker and Cleveland, 1987), an activity in which users draw a rectangular box and select points on a plot by dragging the box around. Brushing over points provides more information about the points, shown in another plot. Linked views and brushing are key features of interactive and dynamic graphics. An illustration of XGobi is also available (http://stat-graphics.org/movies/xgobi.html). The R package, cranvas (Xie et al., 2014), shares a similar spirit with GGobi. GGobi is standalone, and cranvas is available from R. Other standalone interactive data visualization tools include MANET (Unwin et al., 1996) and Mondrian (Theus, 2002).

A command-line interface is useful for running scripts automatically or repeatedly, whereas the strength of a graphical user interface lies in providing the ability to interactively perform statistical data exploration (Unwin and Hofmann, 1999). Graphical user interface designs constitute an integral element of interactive graphics. Interactive visualization software, such as GGobi and cranvas, require users to install cross-platform widget toolkits (e.g., GTK and Qt) on a local computer to create graphical user interfaces that accept user inputs. However, installing such complex toolkits while solving all dependencies is cumbersome for most. These early efforts led to the development of more recent interactive visualization tools that use JavaScript as the front-end, such as Plotly.js, which can be deployed on the web and is accessible from modern web browsers without having to install extra graphic systems.

### Plotly interactive graphing library

Plotly.js is an open-source JavaScript library built on D3.js (Bostock et al., 2011) for performing interactive and dynamic data visualization. This should not be confused with Plotly, the company that maintains Plotly.js. Other programming languages, such as Julia, MATLAB, R, and Python, have their own interfaces to the Plotly.js library, allowing users to create Plotly based interactive graphics without knowledge of web development (i.e., HTML, cascading style sheet, and JavaScript). The R package used to create Plotly graphs is plotly (Sievert, 2020) and users can access all available functionalities of Plotly.js from this package. This allows users to combine the strengths of R (statistical computing) and JavaScript (interactive and dynamic media). The plot_ly function in the **plotly** package maps to the application programming interface of Plotly.js and is the main engine of the plotly R package. With Plotly-based graphics, the default setting incorporates a mouse hover to show additional information about each graphical mark and a mouse click of legends creates switches to highlight or hide the corresponding graphical marks. Additionally, a single call to the function, ggplotly, can easily convert a ggplot object, described under the section, Grammar of graphics, to a Plotly-based interactive graphic.

**Figure 7.**
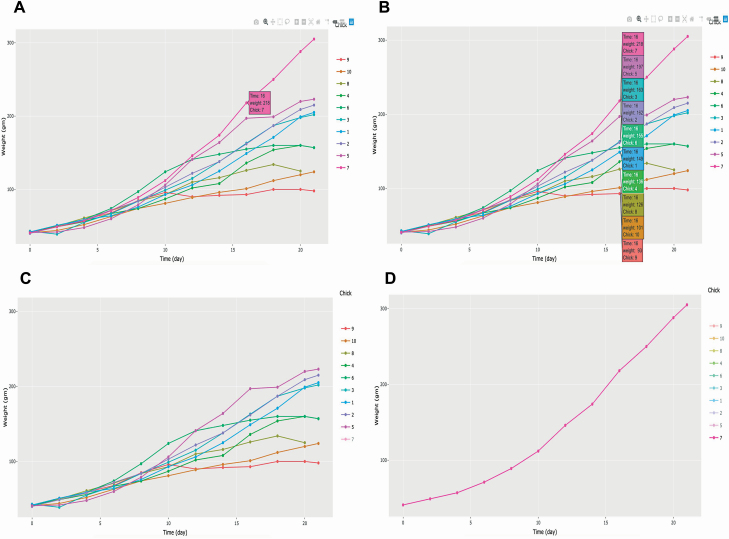
Interactive visualization enabled by the plotly R package. Growth trajectories of 10 chickens over 21 d are shown. A: Displaying chick 7 data at the age of 16 d on hover. B: Comparing weight of 10 chicks at the age of 16 d. C: Single click on the chick 7 legend hides its trace. D: Double-click on the chick 7 legend isolates its trace. The interactive version of this plot is available at http://emitanaka.org/supp/anisci-datavis/#chick-plotly.

To illustrate the capability of the plotly R package, Figure 7 shows line plots with points for the growth trajectories of the first 10 chicks (chick ID 1 to 10) raised on the same diet according to the chick data. Each chick was weighed every 2 d for 21 d after hatching, including the day of hatching and on the 21st day, except for chick 8, who was not weighed on the last day. By default, a mouse hover displays the time (x-axis), weight (y-axis), and chick ID of the closest data point. Figure 7A shows that the weight of chick 7 at 16 d old was 218 g. Displaying all data points at a certain x-axis is possible for comparison purposes by enabling the comparison of data on the hover option, shown at the top right. For example, the lowest weight of a 16-d-old chick was chick 9, who weighed 93 g (Figure 7B). A single-click on the chick 7 legend causes the legend to become partially transparent, concealing its lines and points (Figure 7C). A double-click on the chick 7 legend renders the rest of the legends partially transparent so that the user can isolate its lines and points (Figure 7D). An additional single- or double-click of the chick 7 legend restores the original figure. Through this example, we can see how interactive visualization enables the efficient exploration of data. Additional capabilities easily accessible at the top right corner include the ability to download the plot as a PNG file and to zoom in or out.

**Figure 8.**
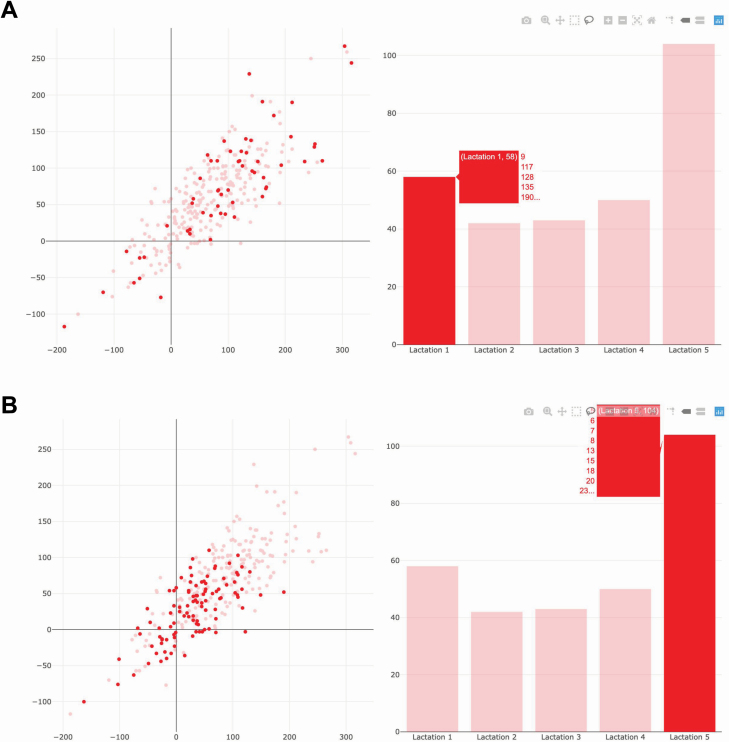
Linking an interactive scatter plot and a bar chart to explore the Jersey data. The scatter plot on the left panel displays the relationship between fat yield deviation (x-axis) and protein yield deviation (y-axis). The bar chart on the right panel shows the frequency of cows classified according to their lactation numbers. A: Hovering on lactation 1 bar highlights lactation 1 cows in the scatter plot. B: Hovering on lactation 5 bar highlights lactation 5 cows in the scatter plot. The interactive version of the multiple linked plots is available at http://emitanaka.org/supp/anisci-datavis/#jersey-plotly.

The plotly package enables users to generate multiple plots with linked interactivity in a web-based format, which is the key feature of GGobi. To illustrate the utility of linked plots, Figure 8 shows a linked scatter plot and a bar chart describing the Jersey cow data. The interactive scatter plot on the left panel displays the relationship between fat yield deviation (x-axis) and protein yield deviation (y-axis). The interactive bar chart on the right panel shows the frequency of cows classified according to their lactation numbers. A mouse hover on lactation 1 bar simultaneously highlights lactation 1 cows in the scatter plot (Figure 8A). Similarly, hovering over lactation 5 bar highlights lactation 5 cows in the scatter plot (Figure 8B). Because the 2 plots are linked, hovering on the data point of cow in the scatter plot also dynamically highlights the corresponding lactation bar according to the cow’s lactation number. As shown in this example, linked views can be used to effectively query data.

### Grammar of interactive graphics for R

The grammar of *interactive* graphics is less mature than the grammar of graphics. There have been several attempts at implementations following the coming of age of the ggplot2 R package (Wickham, 2016). The ggvis R package (Chang and Wickham, 2020) builds on the layering of ggplot, and adds interactivity via mouse over tooltip labeling, or a linked brush which allows selection of subsets in one plot to be broadcast to another. The interactivity is made possible by Vega (Satyanarayan et al., 2016) for rendering graphics on a web browser. The animint2 R package layers interactivity directly on a **ggplot2** plot by translating the plot into JavaScript with a set of standard interactions (Sievert et al., 2019). The cranvas package documented by Xie et al., (2014) illustrates how a scriptable interactive data visualization system might be engineered in R with an appropriate toolkit. The vegawidget (Lyttle and Vega/Vega-Lite Developers 2020) has potential for providing a foundation for scripting interactive graphics. The R package crosstalk (Cheng, 2020) forms the basis of linking data between plot objects such as plotly. This is a space to watch for mature conceptualization and technology to emerge soon.

### Interactive and dynamic tables

A table chart or data table is often used for information presentation and organization. Tables are among the most widely used avenues to present data with rows corresponding to subjects and columns corresponding to variables. Additionally, a table is commonly observed in a data frame structure in R or spreadsheets (e.g., Microsoft Excel and Google Sheets). Some recommended practices for making static spreadsheets more useful were discussed by Broman and Woo (2018). The strength of having interactive and dynamic environments is that it becomes possible to convert a table to an exploratory data visualization. Dynamic sorting in ascending or descending order coupled with a filtering box will facilitate interactive comparative data analysis. An interactive table can be rendered using the DataTables JavaScript library (https://datatables.net/) or its R interface package, DT (Xie et al., 2020), to perform pagination, searching, filtering, and sorting.

### Shiny web application

**Figure 9.**
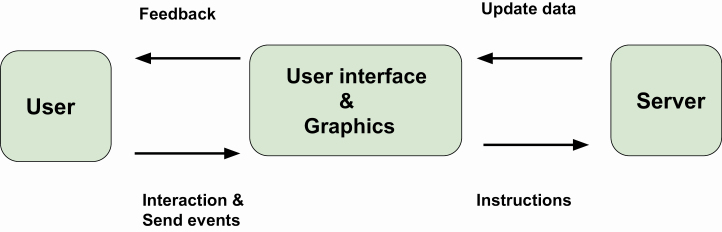
Scheme of Shiny applications. The user interacts or send events to the graphic or control widgets by mouse hovering or clicking. The user interface or graphic process user inputs and pass the code instructions to the server. The server modifies and update the data. The graphic returns feedback to the user.

The R package, shiny (Chang et al., 2020), provides functions for constructing web applications using the R programming language. We refer to the general framework as “Shiny” and any reference to the R package as shiny. This serves as an important bridge for data analysts who are well versed in R but are not familiar with web development. Shiny can be viewed as a generalized unification system that binds input elements (control widgets) and output elements (plots and tables) along with text-based summaries. The Shiny scheme is presented in Figure 9. With Shiny, it is easy to create from R control widgets, such as sliders, radio buttons, check boxes, file upload controls, and text/number boxes so that the user can modify plot or table parameters interactively. Shiny then updates the plots and tables by dynamically responding to the changes received from the input. These functionalities enable the user to modify the underlying parameters and view the results immediately. Rapid visual feedback is key for achieving a satisfactory user experience. Shiny uses reactive programming to make its applications responsive and to reduce computational burdens by updating the output only as necessary. Shiny alone does not offer the capability to create interactive plots or tables. However, users can turn plots and tables into interactive and dynamic tools using the aforementioned Plotly or DataTables or any other R packages in a Shiny application.

**Figure 10.**
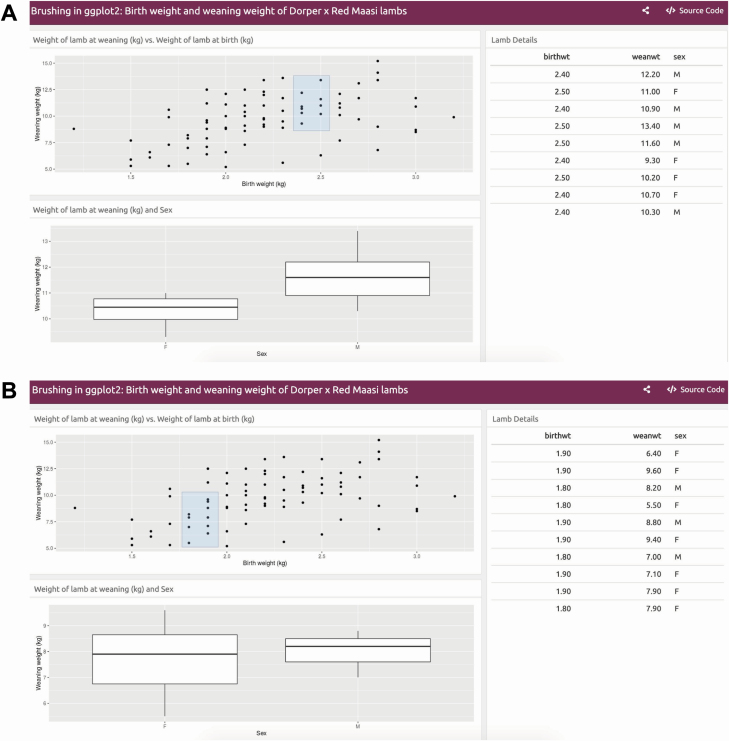
Shiny application for performing brushing in ggplot2 using the Dorper × Red Maasi lamb data. The scatter plot, box plot, and table are linked. (A) The user creates the blue-shaded rectangular box interactively in the scatter plot selecting the data points of interest. The box plot of weaning weight between sex and the table dynamically display information about those selected data points. (B) Brushing or dragging the rectangular box to another group of data points dynamically updates the box plot and the table. The interactive version of the Shiny application is available at https://chikudaisei.shinyapps.io/ggplot2-brushing/.

A Shiny application offers brushing and linked view features. The one shown in Figure 10 was developed using flexdashboard (Iannone et al., 2020) and demonstrates its features using the lamb data. The Shiny application includes the scatter plots of weaning weight vs. birth weight, the box plot of weaning weight between sexes, and the table showing the details of lamb data. First, the user creates a blue-shaded rectangular box interactively using a mouse in the scatter plot and selects the data points of interest (Figure 10A). This triggers dynamic updates of the box plot and the table to display information about only the selected data points. Performing brushing by dragging the rectangular box to another group of data points dynamically flushes the box plot and table (Figure 10B).

### Interactive Manhattan plots

As an extension of the static Manhattan plots, “interactive Manhattan plots”, presents the genome-wide association study results as interactive statistical graphics using the tools described. With interactive Manhattan plots (e.g., ShinyAIM, Hussain et al., 2018), we gain the flexibility to change the cut-off of $-log_{10}(P\text{-}value)$, resulting in a dynamic display of significant genetic markers. A large number of genetic markers under multiple scenarios are usually compared using large-scale or longitudinal genome-wide association studies (Hussain et al., 2018). The combination of direct and indirect manipulation of interactive plots is critical for delivering complete and precise results at large scales by, for example, hovering on genetic markers to provide detailed information, such as marker position, or zooming-in a small genomic region to provide a close-up view with additional details.

### Dynamic documents

Programmatic creation of interactive graphics, such as those using the Plotly or Shiny framework, integrates well with dynamic document. The idea stems from literate programming (Knuth, 1984), where text and code are combined to improve readability. A typical data analysis flow involves writing program code in a file, executing the file, and producing numerical results and figures. This then creates tables according to the numerical results (cut-and-paste) or it causes the figures to download so that the user can insert them into a separate file. With standalone coding, it becomes necessary to repeat the same steps each time manually. However, in a dynamic document, we can unite narratives (e.g., text, figures, and tables) and data analysis (e.g., source code) in a single file. Everything required to execute and recompile the file after updating remains intact. Then, the numerical results, tables, and figures in the single document are automatically updated. Consequently, moving from the source code to the report can be performed in one step. Output formats include HTML, Microsoft Word, PDF, OpenDocument, and rich text format. Document writing frameworks, such as R Markdown (Xie et al., 2018) and R Notebook, coupled with the knitr R package (Xie, 2015) or Jupyter Notebook can generate an interactive HTML graph, provided the output format is HTML. Similarly, an R Markdown document can be made interactive by adding Shiny via the flexdashboard R package (Iannone et al., 2020). Notably, writing a dynamic document promotes its reproducibility (Gentleman, 2005), because the results and the computer code are adjacent in the same file, and the former is automatically generated from the latter (Xie, 2014).

### Educational tools

A web-based multimedia learning tool coupled with an interactive interface have great potential in making materials more accessible or engaging to a wider audience. It has been reported that interactive functionality and 3D modeling enhance spatial thinking (Cohen and Hegarty, 2014). We outline below some educational tools that offer interactive experiences to users in animal science.

Be-Breeder is a Shiny-based online tool designed for teaching basic concepts in population and quantitative genetics actively using interactive statistical graphics (Fritsche-Neto and Matias, 2016; Matias et al., 2019). Users can interactively simulate how allele frequencies, the Hardy–Weinberg equilibrium, genetic drift, selection, additive genetic variance, dominance genetic variance, heterosis, or inbreeding coefficients change while varying the other parameters or keeping the remainder constant. Be-Breeder is capable of performing statistical analyses (e.g., linear mixed models), albeit with limited statistical models, tailored to plant breeding. Some of these applications are also applicable to animal breeding.

The Ruminant Gut Microbe of the Month (Ivey and Myer, 2018) aims to advance the understanding of nutritional microbiology in livestock. The web-based interactive interface designed on the SharePoint platform shows the name of a microbe and its brief overview on the left panel. The main image of the model animal is presented on the right. By hovering a mouse or clicking the hotspots within the main image, pop-up boxes containing detailed descriptions of the microbial function are displayed. The webpage is updated monthly. Different microbes play important roles in the gastrointestinal tracts of animals. Past presented microbial features are available in the archive section.

Images obtained from computed tomography or magnetic resonance imaging that capture anatomy and function of animals are often used for diagnosis and treatment. The Interactive Veterinary Education Tool, mainly implemented in JavaScript, is a web-based e-learning multimedia platform for veterinary teaching, encompassing major livestock species (Xiberta and Boada, 2019). Users can solve exercises and quizzes while exploring 2D images and 3D models interactively. Other interactive e-learning platforms exist, however, many are species specific (El Sharaby et al., 2015; Raffan et al., 2017).

## Computer Vision

Computer vision is a rapidly growing technology. Although it should not be confused with data visualization, because the focus is on automatic analysis of images, not plotting data, it is a potential growth area for animal science research. The methodology involves massive data collection, which requires automatic processing using deep learning (e.g., convolutional neural networks). The data structures generated for computer vision are images, which are converted into a row of a high-dimensional data object for modeling. Data visualization can be helpful in diagnosing and understanding the complex modeling.

Providing suitable animal care is critical for maximizing productivity while ensuring animal welfare and minimizing the environmental footprint. However, labor-based monitoring is intensive and carries a risk of inducing stress to animals or injuries to humans. Precision livestock farming aims to integrate sensor-based technology into daily farm operations to provide continuous real-time monitoring and phenotyping of animals without direct contact (Berckmans, 2017). A recent trend of facilitating the deployment of precision livestock farming has generated new types of data. In particular, a computer vision system accelerates phenotyping by providing non-intrusive measurements of animals with high temporal and spatial resolution. Computer vision or image-based phenotyping obtains phenotypes from video recordings that consist of a sequence of images or frames. Using image-based phenotyping technologies facilitates the automatic collection of phenotypes from large numbers of animals, requiring less human labor while reducing costs, and the evaluation of phenotypes that were previously difficult to measure manually.

**Figure 11.**
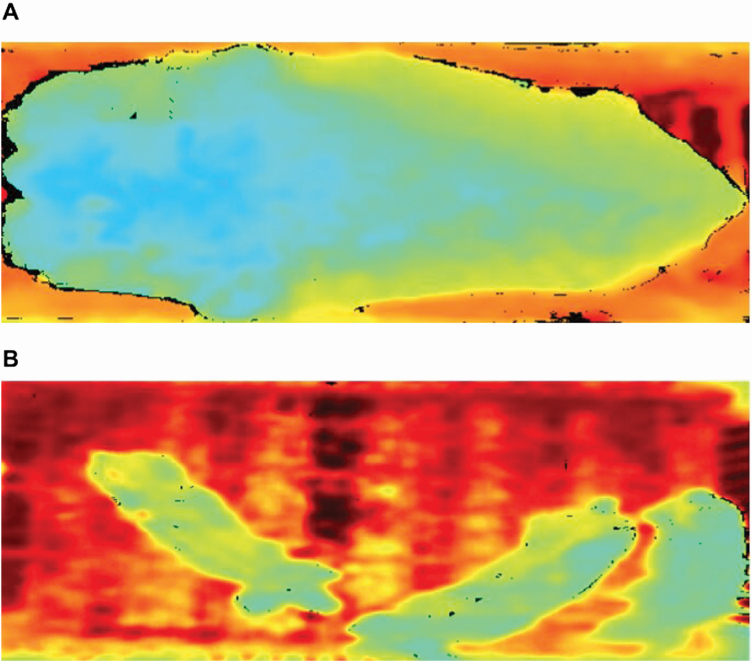
Visualization of a dairy cow (A) and a group of pigs (B) using top-view depth images. Continuously varying heights are indicated by different colors.

Low-cost consumer 3D depth-sensor cameras, such as Microsoft Kinect (Microsoft, Redmond, WA) and Intel RealSense (Intel, Santa Clara, CA), have been used to predict morphological characters and monitor animal behavior (Condotta et al., 2020). A depth-sensor camera positioned at the ceiling visualizes animals in a 3D space by adding height information using the time-of-flight or structured light technology. It returns the distances from the camera to the ground and from the camera to the animal. These 2 distance measures allow users to estimate the height of the animal. It can also be used to monitor whether the animal is lying, sitting, or standing (Lee et al., 2016). Figure 11 shows the depth images of a dairy cow (Figure 11A) and a group of pigs (Figure 11B), where continuously varying heights are indicated by different colors (Yu et al., 2021). From these depth images, we can obtain animal image descriptors, such as length, width, height, and volume. Among the many available image analysis approaches, deep learning algorithms have recently been successfully used in computer vision problems of animal science (Fernandes et al., 2020a). Deep learning does not require tedious image preprocessing steps. Additionally, image processing with deep learning has the potential to become a rapid, reliable, and affordable means of assisting veterinary imaging diagnosis. Li et al., 2020 developed an automated imaging diagnostic tool using deep learning techniques to detect left atrial enlargements on canine thoracic radiographs, reaching the same accuracy achieved by expert veterinary radiologists.

A visual illustration of what a computer vision model sees in an image can be found in the work of Olah et al. (2018). The automatic processing of images is done by breaking the images into many small components and applying numerous filters. To produce an accurate model requires an extensive training sample containing many images and a well-defined response variable but the actual model fitting may be achievable with everyday computing tools and the open-source software, Keras, a Python deep-learning library (Allaire and Chollet, 2020), as described by Chollet and Allaire (2018).

## Visualization in web-based decision support systems

Making human-driven decisions in a timely manner is a challenge for farm management, because complex, big, and heterogeneous data must be processed, particularly those from precision livestock farming. Decision support systems aid practitioners to make informed decisions effectively by aggregating and analyzing multiple sources of information. Visualization of the output from decision support systems promotes communicating suggestions or highlighting uncertainty for decision makers. Van Hertem et al., (2017) argued that the availability of visualization tools was essential for practitioners making data-driven decisions, owing to the complexity of precision livestock farming data. Gutiérrez et al. (2019) provided a topical systematic review of visualization techniques specifically applied to supporting decision-making processes in agriculture. They classified a number of journal articles according to their application areas, targeting end-users, design methodologies, visualization techniques used to communicate data, and usability based on a human–computer interaction perspective. They reported that 2D geo-spatial maps, heatmaps, time-series plots, and histograms were dominantly used as visualization techniques in agriculture.

The development of decision support systems for livestock farming is an ongoing research area (Donnelly et al., 1997; Rotz et al., 1999; Berntsen, 2003; Tedeschi et al., 2004; Cabrera et al., 2005; Meul et al., 2008; Gibbs et al., 2015). In particular, web-based decision support systems can reach a wider range of practitioners, because the sole requirement is a modern web browser. ShinyGPAS (Morota, 2017) is a Shiny-based application that simulates deterministic formulas for the accuracy of genomic predictions (https://chikudaisei.shinyapps.io/shinygpas/). Phenotyping and genotyping of animals constitute a large proportion of expenses in breeding programs. Users can interactively explore achievable prediction accuracy by simultaneously varying potential factors that impact prediction accuracy in deterministic formulas prior to phenotyping and genotyping their own animals. The adjustable parameters include heritability, the number of molecular markers, the proportion of genetic variance explained by the molecular markers, number of animals in a reference set, number of independent chromosome segments underlying a trait, and effective population size. The control widgets are shown on the left panel and the 2D scatterplot of parameter value vs. expected prediction accuracy is displayed on the right panel. ViPER is a decision support tool to assess the microbial risks of pastures from grazing livestock by spatially mapping the *Escherichia coli* load (Oliver et al., 2017). The application is a web-based tool (http://www.nercviper.co.uk/) that supports beef cow, dairy cow, calves, sheep, and lambs from the accumulation of *E. coli* in feces, which vary according to livestock categories. The user can interactively drag and drop animals to the 2D map of agricultural landscapes to adjust grazing numbers and densities. The tool visualizes the expected patterns of *E. coli* loading on each plot spatially and temporally. A heatmap is layered over the plots to indicate alert levels. BeefTracker is a web-based application for monitoring beef cattle production using a graphical pasture interface (Oltjen et al., 2017). The software allows users to quantify the sustainability status of their farm operations by collecting herd data and inventory records.

## Conclusion

In 1987, Becker et al. (1987) stated that interactive and dynamic graphics would be ubiquitous in the future. Although it might have taken longer than they expected, the wide use of such graphics has been enabled mostly because of the advent of web technologies. This trend has also reached animal science disciplines. The strength of interactive graphics is that it enhances human–computer interaction and exploratory data analysis. The tools can be further improved if coupled with the capabilities of linked views and brushing. Both static and interactive and dynamic graphics will continue to be a fundamental part of animal science research. The graphical techniques described here can be applied to visualize the processes or results of mathematical modeling (Tedeschi, 2019). For more information, readers are encouraged to refer to the study by Wilke (2019), Sievert (2020), and Wickham (2020) for information on statistical graphics (https://clauswilke.com/dataviz/), R plotly (https://plotly-r.com/), and Shiny (https://mastering-shiny.org/), respectively. Readers are also advised to take a look at R interface packages for interactive graphing libraries that were not covered in this article, including Leaflet for maps, dygraphs for time-series plots, rbokeh and Highcharter for general purpose graphing, and networkD3 and visNetwork for network visualization.

### Code

All graphics in this paper are produced using the R language (R Core Team, 2020). The code to reproduce these graphs are found at http://emitanaka.org/supp/anisci-datavis/ and also as the supplementary file.

## Supplementary Material

skaa402_suppl_Supplementary_Material
