# Supplementary material for: ASAS-NANP SYMPOSIUM: prospects for interactive and dynamic graphics in the era of data-rich animal science
Source: J Anim Sci. 2021 Feb 24;99(2):skaa402. doi: 10.1093/jas/skaa402 (PMC7904041; doi:10.1093/jas/skaa402)
Supplement: skaa402_suppl_Supplementary_Material [file skaa402_suppl_supplementary_material.html]

Prospects for interactive and dynamic data visualization in the era of data-rich animal science


# Prospects for interactive and dynamic data visualization in the era of data-rich animal science

### SUPPLEMENTARY MATERIAL

#### Gota Morota

#### Hao Cheng

#### Dianne Cook

#### Emi Tanaka

#### 2020-12-10

# About

This is a supplementary material to “*Prospects for interactive and dynamic data visualization in the era of data-rich animal science*”. This page provides all the R code to generate the figures in the aforementioned paper.

Please use the following citation for this paper:

Morota, Cheng, Cook, and Tanaka (2020) Prospects for interactive and dynamic data visualization in the era of data-rich animal science.

And the bibtex is:

```
@Manual{morota2020,
    title = {Prospects for interactive and dynamic data visualization in the era of data-rich animal science},
    author = {Morota, Gota and Cheng, Hao and Cook, Dianne and Tanaka, Emi},
    year = {2020}
}
```

# Data

The quail data is from Sarraude et al. (2020) and the sheep data is from Posbergh and Huson (2020).

See here for the setup code.

```
library(tidyverse)
library(readxl)
library(janitor)
library(colorspace)
library(patchwork)
library(grid)
library(scales)
library(mgcv)
library(mgcViz) # for simulate.gamm
library(genio) # for reading bam file
library(rrBLUP) # for GWAS

knitr::opts_chunk$set(echo = TRUE, 
                      fig.align = "center",
                      #fig.path = "figures/",
                      dev = c("png"))
```

```
quail_df <- read_xlsx("data/Sarraude et al. - THs elevation in Japanese quails - dataset.xlsx",
                      sheet = 3, na = "NA") %>% 
  mutate(motherID = factor(motherID),
         eggID = factor(eggID),
         group = factor(group),
         sex = factor(sex)) %>% 
  # make the label prettier for graphs
  mutate(sex = fct_recode(sex, 
                          "Female" = "F",
                          "Male" = "M"),
         group = fct_recode(group,
                            "T[3]" = "T3",
                            "T[4]" = "T4",
                            "T[3]~T[4]" = "T3T4"))
```

```
bim <- read_bim("data/CPosbergh_USBodySize_217Ewes_500K.bim")
```

```
## Reading: data/CPosbergh_USBodySize_217Ewes_500K.bim
```

```
fam <- read_fam("data/CPosbergh_USBodySize_217Ewes_500K.fam")
```

```
## Reading: data/CPosbergh_USBodySize_217Ewes_500K.fam
```

```
# marker by genotype matrix 
X <- read_bed("data/CPosbergh_USBodySize_217Ewes_500K.bed", bim$id, fam$id)
```

```
## Reading: data/CPosbergh_USBodySize_217Ewes_500K.bed
```

```
geno <- as.data.frame(X - 1) %>% 
  rownames_to_column('marker') %>% 
  left_join(select(bim, id, chr, pos), by = c("marker" = "id")) %>% 
  select(marker, chr, pos, everything()) 

sheep_df <- read_csv("data/Posbergh_616ewes_BodyMeasures.csv") %>% 
  clean_names() %>% 
  filter(animal_id %in% colnames(geno)) %>% 
  as.data.frame()
```

```
## 
## ── Column specification ────────────────────────────────────────────────────────
## cols(
##   .default = col_double(),
##   Breed = col_character()
## )
## ℹ Use `spec()` for the full column specifications.
```

# Figure 1

```
g1 <- ggplot(quail_df, aes(day, mass, color = group)) +
  geom_line(aes(group = eggID)) + 
  facet_wrap(~sex) +
  labs(x = "Days since hatching", 
       y = "Body mass (g)", 
       color = "Treatment", 
       tag = "A") +
  scale_color_discrete_qualitative(labels = str2expression,
                                   limits = c("CO", "T[3]", "T[4]", "T[3]~T[4]")) +
  theme(legend.text.align = 0,
        plot.tag = element_text(face = "bold", size = 18))

g2 <- ggplot(quail_df, aes(day, mass, color = group)) +
  stat_summary(fun = mean, geom = "line") + 
  stat_summary(fun.data = mean_se, geom = "pointrange", fatten = 2) +
  facet_wrap(~sex) +
  labs(x = "Days since hatching", 
       y = "Body mass (g)", 
       color = "Treatment", 
       tag = "B") +
  scale_color_discrete_qualitative(labels = str2expression,
                                   limits = c("CO", "T[3]", "T[4]", "T[3]~T[4]")) +
  theme(legend.text.align = 0,
        plot.tag = element_text(face = "bold", size = 18))

g1 / g2
```

# Figure 2

```
ggplot(quail_df, aes(day, mass)) +
  geom_line(data = select(quail_df, -c(sex, group)), 
            aes(group = eggID), color = "gray") + 
  geom_line(aes(group = eggID)) + 
  facet_grid(sex ~ group,
             labeller = label_parsed) +
  labs(x = "Days since hatching", y = "Body mass (g)")
```

# Figure 3

```
fit <- gamm(mass ~ group + sex + s(day, by = sex) + s(motherID, bs = "re"),
                correlation = corARMA(form = ~ 1 | day, p = 1, q = 1),
                data = quail_df)

pred_df <- quail_df %>% 
  mutate(pred = predict(fit$gam),
         .resid = residuals(fit$gam))
```

```
set.seed(2020)
pred_df %>% 
  # pack the data up for each egg
  group_by(eggID, group, sex) %>% 
  nest() %>% 
  # sample 1 for each treatment x sex group
  group_by(group, sex) %>% 
  sample_n(1) %>% 
  # unpack 
  unnest(data) %>% 
  # plot
  ggplot(aes(day, mass, group = eggID)) +
  geom_text(aes(label = eggID), size = 15, alpha = 0.05, color = "gray",
            x = mean(range(quail_df$day)), y = mean(range(quail_df$mass))) + 
  geom_point() + 
  geom_line() + 
  geom_line(aes(y = pred), color = "red") +
  facet_grid(sex ~ group, labeller = label_parsed) +
  labs(x = "Days since hatching", y = "Body mass (g)")
```

# Figure 4

This is the residual plot which get embeded into a random position in the lineup shown next.

```
gres <- pred_df %>% 
  ggplot(aes(day, .resid)) +
  geom_point() + 
  geom_hline(yintercept = 0, linetype = "dashed") +
  labs(x = "Days since hatching", y = "Residuals")
gres
```

```
set.seed(2020)
m <- 6 # the size of the lineup
pos <- sample(m, size = 1)  # the position of the data plot position

lineup <- map_dfr(1:m, ~{
  tmp_df <- pred_df
  if(.x!=pos) {
    tmp_df <- tmp_df %>% 
      mutate(mass = simulate.gam(fit$gam)[1,],
             .sample = .x)
    tmp_fit <- gamm(mass ~ group + sex + s(day, by = sex) + s(motherID, bs = "re"),
                    correlation = corARMA(form = ~ 1 | day, p = 1, q = 1),
                    data = tmp_df)
    tmp_df <- tmp_df %>% 
      mutate(.resid = residuals(tmp_fit$gam))
  }
  mutate(tmp_df, .sample = .x)
})

gres %+% lineup + 
  facet_wrap(~.sample, nrow = 2) +
  theme(axis.text = element_blank(),
        axis.title = element_blank())
```

# Figure 5

```
out_df <- GWAS(pheno = select(sheep_df, animal_id, height_withers),
               geno = geno, min.MAF = 0.05) %>% 
  mutate(chr = as.numeric(chr))
```

```
## [1] "GWAS for trait: height_withers"
## [1] "Variance components estimated. Testing markers."
```

```
pvalcutoff1 <- -log10(1e-7) 
pvalcutoff2 <- -log10(1e-5)

g1 <- out_df %>% 
  ggplot(aes(pos, height_withers)) +
  geom_point(aes(color = chr %% 2 == 0)) + 
  facet_grid(. ~ chr, scales = "free_x", space = "free_x", switch = "x") +
  theme(panel.spacing.x = unit(0, "lines")) +
  scale_color_manual(values = c("black", "gray")) +
  guides(color = FALSE) +
  scale_y_continuous(expand = c(0, 0), 
                     limits = c(0, max(out_df$height_withers) + 1.5),
                     breaks = seq(0, max(out_df$height_withers), by = 2.5)) +
  theme(axis.text.x = element_blank(),
        axis.ticks.length.x = unit(0, "lines"),
        panel.background = element_rect(fill = "transparent"),
        plot.tag = element_text(face = "bold", size = 18),
        panel.grid = element_blank()) + 
  labs(x = "Chromosome", y = expression(-log[10]("p-value")),
       tag = "A") 

g2 <- g1 + geom_hline(yintercept = c(pvalcutoff1, pvalcutoff2), linetype = "dashed") +
  geom_point(data = filter(out_df, height_withers > pvalcutoff1), 
             color = "blue", size = 2) +
  geom_text(data = filter(out_df, height_withers > pvalcutoff1), 
                           aes(x = pos - 20000000, 
                               label = paste0("Chr: ", chr, "\nPos: ", comma(pos, big.mark = " "), "bp")),
                           nudge_y = 0.9, hjust = "left") +
  coord_cartesian(clip = "off") +
  labs(tag = "B")

g1 / g2
```

# Figure 7

```
g <- ChickWeight %>% 
  filter(Chick %in% 1:10) %>% 
  mutate(Chick = factor(Chick, ordered = FALSE)) %>% 
  ggplot(aes(Time, weight, color = Chick)) +
  geom_point() + 
  geom_line() +
  labs(x = "Time (day)", y = "Weight (gm)")
```

```
library(plotly)
ggplotly(g)
```

Hover over the points with the mouse to view information of each point.

# Figure 8

```
data(Jersey, package = "brnn")
df <- pheno %>% 
  rename(   Milk = yield_devMilk,
             Fat = yield_devFat,
         Protein = yield_devProt) %>% 
  mutate(num_lactat = paste("Lactation", num_lactat))
```

```
base <- highlight_key(df) %>% 
  plot_ly(color = I("red"), showlegend = FALSE)

subplot(
  add_markers(base, x = ~Fat, y = ~Protein),
  add_histogram(base, x = ~num_lactat)
) %>%
  layout(barmode = "overlay") %>%
  highlight( "plotly_hover")
```

# Figure 10

The interactive web application can be found here. The source code to generate the app is available by clicking the “Source Code” button on the top right corner of the Shiny application.

# Session Information

This document is made using R Markdown and R packages as shown in the session information below.

Session Information

```
sessioninfo::session_info()
```

```
## ─ Session info ───────────────────────────────────────────────────────────────
##  setting  value                       
##  version  R version 4.0.1 (2020-06-06)
##  os       macOS Catalina 10.15.7      
##  system   x86_64, darwin17.0          
##  ui       X11                         
##  language (EN)                        
##  collate  en_AU.UTF-8                 
##  ctype    en_AU.UTF-8                 
##  tz       Australia/Melbourne         
##  date     2020-12-10                  
## 
## ─ Packages ───────────────────────────────────────────────────────────────────
##  package          * version    date       lib source                           
##  assertthat         0.2.1      2019-03-21 [2] CRAN (R 4.0.0)                   
##  backports          1.2.0      2020-11-02 [1] CRAN (R 4.0.2)                   
##  boot               1.3-25     2020-04-26 [2] CRAN (R 4.0.1)                   
##  broom              0.7.2      2020-10-20 [1] CRAN (R 4.0.2)                   
##  cellranger         1.1.0      2016-07-27 [2] CRAN (R 4.0.0)                   
##  cli                2.2.0      2020-11-20 [1] CRAN (R 4.0.1)                   
##  codetools          0.2-18     2020-11-04 [2] CRAN (R 4.0.1)                   
##  colorspace       * 2.0-0      2020-11-11 [1] CRAN (R 4.0.2)                   
##  crayon             1.3.4      2017-09-16 [2] CRAN (R 4.0.0)                   
##  crosstalk          1.1.0.1    2020-03-13 [1] CRAN (R 4.0.2)                   
##  data.table         1.13.2     2020-10-19 [2] CRAN (R 4.0.2)                   
##  DBI                1.1.0      2019-12-15 [1] CRAN (R 4.0.2)                   
##  dbplyr             2.0.0      2020-11-03 [1] CRAN (R 4.0.2)                   
##  digest             0.6.27     2020-10-24 [1] CRAN (R 4.0.2)                   
##  doParallel         1.0.16     2020-10-16 [1] CRAN (R 4.0.2)                   
##  dplyr            * 1.0.2      2020-08-18 [1] CRAN (R 4.0.2)                   
##  ellipsis           0.3.1      2020-05-15 [2] CRAN (R 4.0.0)                   
##  evaluate           0.14       2019-05-28 [2] CRAN (R 4.0.0)                   
##  fansi              0.4.1      2020-01-08 [2] CRAN (R 4.0.0)                   
##  farver             2.0.3.9000 2020-07-24 [1] Github (thomasp85/farver@f1bcb56)
##  fastmap            1.0.1      2019-10-08 [2] CRAN (R 4.0.0)                   
##  forcats          * 0.5.0      2020-03-01 [2] CRAN (R 4.0.0)                   
##  foreach            1.5.1      2020-10-15 [1] CRAN (R 4.0.2)                   
##  fs                 1.5.0      2020-07-31 [1] CRAN (R 4.0.2)                   
##  gamm4              0.2-6      2020-04-03 [1] CRAN (R 4.0.2)                   
##  generics           0.1.0      2020-10-31 [2] CRAN (R 4.0.2)                   
##  genio            * 1.0.12     2019-12-17 [1] CRAN (R 4.0.2)                   
##  GGally             2.0.0      2020-06-06 [1] CRAN (R 4.0.2)                   
##  ggplot2          * 3.3.2      2020-06-19 [1] CRAN (R 4.0.2)                   
##  glue               1.4.2      2020-08-27 [1] CRAN (R 4.0.2)                   
##  gridExtra          2.3        2017-09-09 [2] CRAN (R 4.0.0)                   
##  gtable             0.3.0      2019-03-25 [2] CRAN (R 4.0.0)                   
##  haven              2.3.1      2020-06-01 [2] CRAN (R 4.0.0)                   
##  hms                0.5.3      2020-01-08 [2] CRAN (R 4.0.0)                   
##  htmltools          0.5.0      2020-06-16 [1] CRAN (R 4.0.2)                   
##  htmlwidgets        1.5.2      2020-10-03 [1] CRAN (R 4.0.2)                   
##  httpuv             1.5.4      2020-06-06 [2] CRAN (R 4.0.1)                   
##  httr               1.4.2      2020-07-20 [1] CRAN (R 4.0.2)                   
##  iterators          1.0.13     2020-10-15 [1] CRAN (R 4.0.2)                   
##  janitor          * 2.0.1      2020-04-12 [2] CRAN (R 4.0.0)                   
##  jsonlite           1.7.1      2020-09-07 [1] CRAN (R 4.0.2)                   
##  KernSmooth         2.23-18    2020-10-29 [2] CRAN (R 4.0.2)                   
##  knitr              1.30       2020-09-22 [1] CRAN (R 4.0.2)                   
##  labeling           0.4.2      2020-10-20 [1] CRAN (R 4.0.2)                   
##  later              1.1.0.1    2020-06-05 [2] CRAN (R 4.0.1)                   
##  lattice            0.20-41    2020-04-02 [2] CRAN (R 4.0.1)                   
##  lazyeval           0.2.2      2019-03-15 [2] CRAN (R 4.0.0)                   
##  lifecycle          0.2.0      2020-03-06 [1] CRAN (R 4.0.0)                   
##  lme4               1.1-25     2020-10-23 [1] CRAN (R 4.0.2)                   
##  lubridate          1.7.9      2020-06-08 [2] CRAN (R 4.0.1)                   
##  magrittr           2.0.1      2020-11-17 [1] CRAN (R 4.0.2)                   
##  manipulateWidget   0.10.1     2020-02-24 [1] CRAN (R 4.0.2)                   
##  MASS               7.3-53     2020-09-09 [1] CRAN (R 4.0.2)                   
##  Matrix             1.2-18     2019-11-27 [2] CRAN (R 4.0.1)                   
##  matrixStats        0.57.0     2020-09-25 [1] CRAN (R 4.0.2)                   
##  mgcv             * 1.8-33     2020-08-27 [2] CRAN (R 4.0.2)                   
##  mgcViz           * 0.1.6      2020-03-04 [1] CRAN (R 4.0.2)                   
##  mime               0.9        2020-02-04 [2] CRAN (R 4.0.0)                   
##  miniUI             0.1.1.1    2018-05-18 [1] CRAN (R 4.0.0)                   
##  minqa              1.2.4      2014-10-09 [2] CRAN (R 4.0.0)                   
##  modelr             0.1.8      2020-05-19 [2] CRAN (R 4.0.0)                   
##  munsell            0.5.0      2018-06-12 [2] CRAN (R 4.0.0)                   
##  nlme             * 3.1-150    2020-10-24 [2] CRAN (R 4.0.2)                   
##  nloptr             1.2.2.2    2020-07-02 [2] CRAN (R 4.0.1)                   
##  patchwork        * 1.0.1      2020-06-22 [1] CRAN (R 4.0.2)                   
##  pillar             1.4.7      2020-11-20 [1] CRAN (R 4.0.1)                   
##  pkgconfig          2.0.3      2019-09-22 [2] CRAN (R 4.0.0)                   
##  plotly           * 4.9.2.1    2020-04-04 [1] CRAN (R 4.0.2)                   
##  plyr               1.8.6      2020-03-03 [2] CRAN (R 4.0.0)                   
##  promises           1.1.1      2020-06-09 [1] CRAN (R 4.0.0)                   
##  purrr            * 0.3.4      2020-04-17 [2] CRAN (R 4.0.0)                   
##  qgam             * 1.3.2      2020-02-03 [1] CRAN (R 4.0.2)                   
##  R6                 2.5.0      2020-10-28 [1] CRAN (R 4.0.2)                   
##  RColorBrewer       1.1-2      2014-12-07 [1] CRAN (R 4.0.1)                   
##  Rcpp               1.0.5      2020-07-06 [1] CRAN (R 4.0.0)                   
##  readr            * 1.4.0      2020-10-05 [2] CRAN (R 4.0.2)                   
##  readxl           * 1.3.1      2019-03-13 [2] CRAN (R 4.0.0)                   
##  reprex             0.3.0.9001 2020-08-08 [1] Github (tidyverse/reprex@9594ee9)
##  reshape            0.8.8      2018-10-23 [1] CRAN (R 4.0.2)                   
##  rgl              * 0.100.54   2020-04-14 [1] CRAN (R 4.0.2)                   
##  rlang              0.4.8      2020-10-08 [1] CRAN (R 4.0.2)                   
##  rmarkdown          2.5        2020-10-21 [1] CRAN (R 4.0.1)                   
##  rrBLUP           * 4.6.1      2019-12-18 [1] CRAN (R 4.0.0)                   
##  rstudioapi         0.13       2020-11-12 [1] CRAN (R 4.0.1)                   
##  rvest              0.3.6      2020-07-25 [1] CRAN (R 4.0.2)                   
##  scales           * 1.1.1      2020-05-11 [2] CRAN (R 4.0.0)                   
##  sessioninfo        1.1.1      2018-11-05 [2] CRAN (R 4.0.0)                   
##  shiny              1.5.0      2020-06-23 [1] CRAN (R 4.0.2)                   
##  snakecase          0.11.0     2019-05-25 [2] CRAN (R 4.0.0)                   
##  statmod            1.4.35     2020-10-19 [2] CRAN (R 4.0.2)                   
##  stringi            1.5.3      2020-09-09 [2] CRAN (R 4.0.2)                   
##  stringr          * 1.4.0      2019-02-10 [2] CRAN (R 4.0.0)                   
##  tibble           * 3.0.4.9000 2020-11-26 [1] Github (tidyverse/tibble@9eeef4d)
##  tidyr            * 1.1.2      2020-08-27 [1] CRAN (R 4.0.2)                   
##  tidyselect         1.1.0      2020-05-11 [2] CRAN (R 4.0.0)                   
##  tidyverse        * 1.3.0      2019-11-21 [1] CRAN (R 4.0.2)                   
##  vctrs              0.3.5.9000 2020-11-26 [1] Github (r-lib/vctrs@957baf7)     
##  viridis            0.5.1      2018-03-29 [2] CRAN (R 4.0.0)                   
##  viridisLite        0.3.0      2018-02-01 [2] CRAN (R 4.0.0)                   
##  webshot            0.5.2      2019-11-22 [2] CRAN (R 4.0.0)                   
##  withr              2.3.0      2020-09-22 [1] CRAN (R 4.0.2)                   
##  xfun               0.19       2020-10-30 [1] CRAN (R 4.0.2)                   
##  xml2               1.3.2      2020-04-23 [2] CRAN (R 4.0.0)                   
##  xtable             1.8-4      2019-04-21 [2] CRAN (R 4.0.0)                   
##  yaml               2.2.1      2020-02-01 [1] CRAN (R 4.0.2)                   
## 
## [1] /Users/etan0038/Library/R/4.0/library
## [2] /Library/Frameworks/R.framework/Versions/4.0/Resources/library
```

# References

Posbergh, C J, and H J Huson. 2020. “All Sheeps and Sizes: A Genetic Investigation of Mature Body Sizea Cross Sheep Breeds Reveals a Polygenic Nature.” *Animal Genetics*. https://doi.org/10.1111/age.13016.

Sarraude, Tom, Bin-Yan Hsu, Ton Groothuis, and Suvi Ruuskanen. 2020. “Dataset of prenatal thyroid hormones manipulation in Japanese quails.” Zenodo. https://doi.org/10.5281/zenodo.3741711.
